# Risk factors and clinical analysis of peripherally inserted central catheter-related fungal colonization in premature infants

**DOI:** 10.1038/s41598-021-00120-0

**Published:** 2021-10-22

**Authors:** Lingping Zhang, Liu Yang, Wenbin Dong, Xingling Liu, Xiaoping Lei, Lianyu Zhang

**Affiliations:** 1 School of Nursing, Health Science Centre, Xi’an Jiaotong University, Xi’an, 710061 Shaanxi China; 2 Clinical Nursing Research Institute, Affiliated Hospital of Southwest Medical University, Luzhou, 646000 Sichuan China; 3 School of Nursing, Southwest Medical University, Luzhou, 646000 Sichuan China; 4 Devision of Newborn Medicine, Department of Pediatrics, Affiliated Hospital of Southwest Medical University, Luzhou, 646000 Sichuan China

**Keywords:** Paediatric research, Preterm birth

## Abstract

We aimed to analyze the risk factors of positive peripherally inserted central catheter (PICC)-related fungal colonization in preterm infants. This retrospective study collected data from 2018 to 2020. The enrolled infants who underwent PICC insertion were born at < 32 weeks’ gestation or birth weight < 1500 g. The demographics, PICC-related characteristics, and treatment information were collected. Univariate and multivariate analyses were performed to investigate risk factors for PICC-related fungal colonization. The receiver operating characteristic (ROC) curve was used to determine the optimal cut-off values for the duration of antibiotics and parenteral nutrition. In total, 124 premature infants underwent PICC insertion. Among them, 19 patients had positive results of fungi on the PICC tips. The duration of antibiotics (odds ratio [OR] 1.16, 95% confidence interval [CI] 1.02–1.31), parenteral nutrition infusion (OR 1.27, 95% CI 1.05–1.54), and postnatal glucocorticoid exposure (OR 9.48, 95% CI 1.06–84.98) were independent risk factors for fungal colonization in PICCs. The ROC curves showed that the risk increased after 15 days of antibiotic use and 28 days of parenteral nutrition infusion. Appropriate clinical management should be used to prevent fungal colonization and fungemia.

## Introduction

Peripherally inserted central catheters (PICCs) are widely used in neonatal intensive care units (NICUs), especially for the treatment of extremely preterm infants and very low birth weight (VLBW) infants to provide secure venous access or safe administration of hyperosmolar solutions^1^. However, the use of PICCs increases the risk of central line-associated bloodstream infection (CLABSI), one of the most common nosocomial infections related to PICCs^2,3^. CLABSI has been associated with several life-threatening complications, including necrotizing enterocolitis, intraventricular hemorrhage, bronchopulmonary dysplasia, and retinopathy of prematurity, extended hospital stay, and increased mortality or morbidity of premature infants^1,4^. Bacteria were the most important pathogen of CLABSI^5^. However, in premature infants, an increasing trend has been shown in fungal infections^6^, and the fungi were another common pathogen of CLABSI^7,8^.

Fungal infection is a severe complication during PICC placement and is often fatal in very premature infants^9^. The colonization of fungi in the catheters is the onset step of PICC-related fungal infections^10,11^. Invasive *Candida* infections can be prevented with antifungal prophylaxis targeted when the central venous catheter is in place in preterm infants^12–14^. However, we still have observed a high positive fungal culture from the PICC tips in our NICU over the past few years. Thus, in the present study, we aimed to explore the risk factors of PICC-related positive catheter tip cultures in very premature infants.

## Results

### Demographics

During the study period, 124 premature infants underwent PICC insertion. Among them, 21 patients were excluded for the following reasons: 1 had accidental detachment, 5 had an ectopic catheter, 6 had the catheter inserted twice, 9 died within the first week of life. The remaining 103 cases of catheter tips were cultured, of whom 28 patients were positive including 19 for fungi and 9 for bacteria, and 75 cases were negative. The most commonly isolated microorganism was fungi (67.9%, 19/28), especially *Candida albicans* and *Candida glabrata*. The followed positive pathogen was bacteria (32.1%, 9/28) (Table 1). None of the blood cultures obtained during the time of the catheter tip cultures showed positive results.

**Table 1:** Microorganisms cultured from the catheter tip (n = 28).

Microorganism	Year			
	2018	2019	2020	Total
**Gram-positive bacteria**				
*Streptococcus viridans*	1	–	–	1 (3.6%)
**Gram-negative bacteria**				
*Pseudomonas aeruginosa*	–	1	5	6 (21.4%)
*Morganella morganii*	–	–	1	1 (3.6%)
*Acinetobacter haemolyticus*	1	–	–	1 (3.6%)
**Fungi**				
*Candida albicans*	5	4	4	13 (46.4%)
*Candida glabrata*	2	2	2	6 (21.4%)

### Univariate analysis of fungal colonization at peripherally inserted central catheter tips

We compared the medical parameters between positive (n = 19) and negative cases (n = 75) for fungal colonization. As shown in Table 2, the positive fungal colonized PICC group had a significantly longer duration of PICC dwelling, antibiotic use, parenteral nutrition (PN) infusion, and postnatal glucocorticoid exposure than the negative fungal colonized PICC group.

**Table 2:** Univariate analysis of fungal colonization at the catheter tips.

Parameter	Total (n = 94)	Positive (n = 19)	Negative (n = 75)	*χ*^2^*/t/Z*	*p*
**Demographic**					
**Sex (n)**				0.00*	0.96
Male	50	10 (52.6%)	40 (53.3%)		
Female	44	9 (47.4%)	35 (46.7%)		
Gestational age (weeks)	29.5 (28.3, 31.4)	29 (27.6, 31.4)	29.6 (28.4, 31.4)	1.05^◆^	0.29
Birth weight (grams)	1224 ± 190	1270 ± 192	1213 ± 189	1.18^★^	0.24
**Mode of delivery (n)**				0.52*	0.47
Vaginal	68	15 (78.9%)	53 (70.7%)		
Caesarean section	26	4 (21.1%)	22 (29.3%)		
**Assisted reproductive technology (n)**				0.08*	0.78
Yes	13	3 (15.8%)	10 (13.3%)		
No	81	16 (84.2%)	65 (86.7%)		
**Twin (n)**				0.18*	0.67
Yes	26	6 (31.6%)	20 (26.7%)		
No	68	13 (68.4%)	55 (73.3%)		
**Premature rupture of membranes (n)**				2.20*	0.14
Yes	22	2 (10.5%)	20 (26.7%)		
No	72	17 (89.5%)	55 (73.3%)		
**PICC-related characteristic**					
Age at catheter insertion (days)	4 (2, 6)	4 (2, 6)	4 (2, 6)	0.31^◆^	0.75
PICC dwelling time (days)	30 (24, 35.3)	37 (33, 44)	28 (22, 33)	4.50^◆^	**< *0.001***
**Tip position (n)**				0.00*	0.99
Superior vena cava	89	18 (94.7%)	71 (94.7%)		
Inferior vena cava	5	1 (5.3%)	4 (5.3%)		
**Treatment**					
Duration of antibiotic (days)	11 (7.75, 20)	22 (20, 29)	10 (5, 15)	5.71^◆^	**< *0.001***
Duration of PN (days)	27 (20, 31.3)	35 (31, 39)	24 (18, 30)	5.41^◆^	**< *0.001***
**Postnatal glucocorticoid exposure (n)**				18.96*	**< *0.001***
Yes	38	16 (84.2%)	22 (29.3%)		
No	56	3 (15.8%)	53 (70.7%)		

*Chi-square test, ^★^*t* test, and ^◆^rank-sum test.

*PICC* peripherally inserted central catheter, *PN* parenteral nutrition.

### Multivariate logistic regression analysis of fungal colonization at peripherally inserted central catheter tips

Multivariate logistic regression analysis was performed to identify the risk factors for fungal colonization. As shown in Table 3, all those factors increased the risk of fungal colonization. Logistic analyses demonstrated that with each additional day of antibiotic use and PN infusion, the risk of fungal colonization in PICC tips was increased by 16% and 27%, respectively. Postnatal exposure to the glucocorticoid also increased the risk by 9.48 times.

**Table 3:** Logistic regression analysis for fungal colonization at the catheter tips.

Parameter	OR	95% CI	Adjusted OR	95% CI
PICC dwelling time (by each day)	1.21	1.09, 1.35	1.07	0.93, 1.23
Antibiotic use (by each day)	1.23	1.11, 1.35	1.16	1.02, 1.31
PN use (by each day)	1.35	1.16, 1.58	1.27	1.05, 1.54
Postnatal glucocorticoid exposure (yes or no)	12.91	3.37, 49.51	9.48	1.06, 84.98

The duration of PN and PICC dwelling time were entered into the logistic regression model only one parameter at a time.

*OR* odds ratio, *CI* confidence interval, *PICC* peripherally inserted central catheter,* PN* parenteral nutrition.

### Optimal cut-off values for the duration of antibiotics and parenteral nutrition

**Figure 1:**
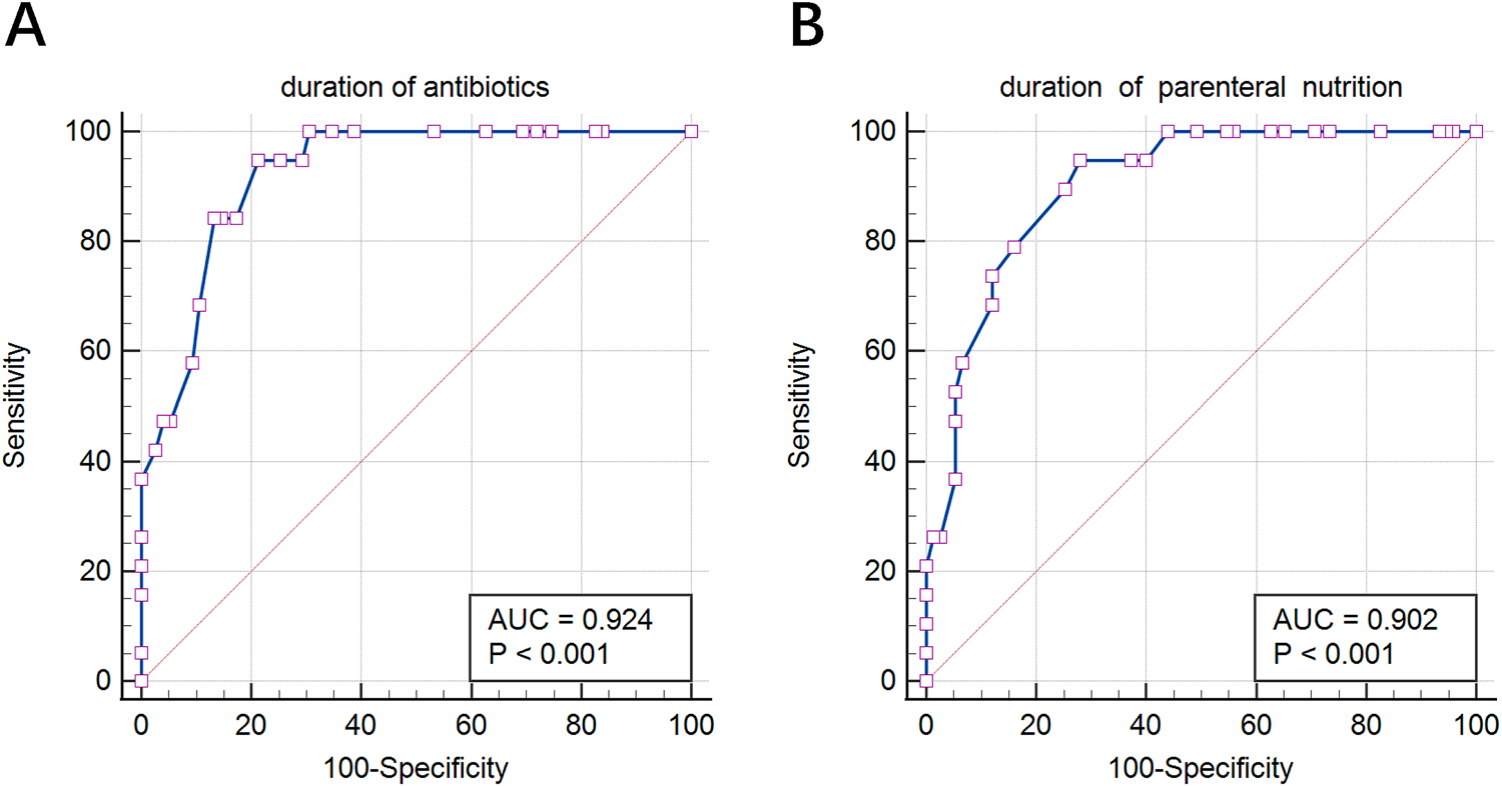
Receiver operating characteristic curve for the duration of antibiotics (**A**) and parenteral nutrition (**B**). AUC, area under the receiver operating characteristic curve.

The calculated optimal cut-off values for the duration of antibiotics and PN were 15 days and 28 days, respectively. Both the area under the receiver operating characteristic (ROC) curve values were larger than 0.9, which showed a high diagnostic value (Fig. 1). The Youden index, sensitivity, and specificity are shown in Table 4.

**Table 4:** Optimal cut-off value for the duration of antibiotics and PN.

	Duration of antibiotics	Duration of PN
Youden index	0.73	0.67
Criterion (days)	> 15	> 28
Sensitivity	94.7	94.7
Specificity	78.6	72.0

*PN* parenteral nutrition.

## Discussion

In recent decades, the incidence of premature infants has increased. Very premature infants or VLBW infants have had long hospitalizations. Because of poor peripheral vascular conditions, the use of PICC has increased substantially. However, exogenous catheters are associated with an increased risk of CLABSI^15,16^. Previous studies have shown that coagulase-negative *Staphylococcus* was the predominant pathogen of CLABSI in the NICU^17,18^. However, recently, it seems that the proportion of fungi has increased in PICC-associated bloodstream infections^19^. A previous study^20^ showed that the distribution of neonatal fungal culture-positive strains has gradually shifted to non-*C. albicans* strains. *Candida parapsilosis* has emerged as a main cause of candidemia worldwide among non-*albicans* species^21^. However, the composition of pathogens varies by hospital, with *C. albicans* ranked first, followed by *C. glabrata*; no case of *C. parapsilosis* colonization was found in the present study.

Nosocomial infections caused by *Candida* species are a public health problem because of their severity and high morbidity and mortality rates^22^. To prevent the occurrence of PICC-associated fungemia, the risk factors of fungal colonization at PICC tips were analyzed in the current study. Multivariate logistic regression analysis identified significant risk factors: the duration of antibiotics, PN infusion, and postnatal glucocorticoid exposure.

A high proportion (66/94, 70%) of premature infants were suspected to have early-onset sepsis and were empirically treated with antibiotics if any of the following were present within 3 days of age: abnormal clinical manifestations, the mother had chorioamnionitis, and premature rupture of membranes exceeded 18 h^23^. However, the long-term use of broad-spectrum antibiotics will cause a flora imbalance in the body, induce drug-resistant bacteria, cause refractory sepsis, and even trigger the outbreak of nosocomial infection^24^. In addition, long-term use of antibiotics carries various side effects, such as damage to liver and kidney function, further decline of immunity, and increased probability of fungal infection^25^. Therefore, risk assessment methods should be used to optimize early antibiotic practice^26^. Antibiotics should also be withdrawn as soon as possible without indication to reduce fungi colonization. In particular, clinicians have a high suspicion for fungal infections in infants who have been receiving antibiotics for a long time. Especially, if the duration of antibiotics exceeds 15 days, the infant’s risk of fungal colonization will be greatly increased.

Furthermore, the present study determined that the duration of PN was a risk factor for fungal colonization at PICC tips. Fungi, especially *Candida* spp., which are notoriously adept at forming drug-resistant biofilm structures, are strongly adherent to surfaces and easily colonize in the PICC catheter. PN solutions contain electrolytes, micronutrients, and the macronutrients dextrose, amino acids, and lipid emulsions. Hypertonic glucose in the PN solutions is ideal for *Candida* growth, while fat emulsions are suitable for the growth of various microorganisms^27^. These conditions increase the risk of fungal colonization in PICCs.

We determined that the optimal cut-off value for the duration of PN was 28 days. However, in clinical practice, it is common for extremely preterm infants to need PN for more than 28 days, which means that the PICC is still required. Additionally, based on other literature^28^, replacing the PICC with one at a new site can be considered to reduce the risk of CLABSI. On the other hand, it has also been suggested that clinicians improve nutritional management and use measures including breastfeeding, early micro-feeding, and oral massage to improve infants’ feeding tolerance and reduce the duration of PN^29–31^.

Glucocorticoids are widely used in very preterm infants to minimise the magnitude and duration of ventilatory support and decrease pulmonary morbidity. At the same time, glucocorticoids can increase the risk of fungal sepsis^32^. Our study showed similar results that postnatal glucocorticoid exposure increased the risk of catheter fungal colonization by 9.48 times. This might be related to dexamethasone therapy, which has side effects on neutrophils and lymphocytes and thus increases the risk of fungal infection in preterm infants^32^. Hence, strict guidelines should be implemented for the use of glucocorticoids in clinical practice. Patients should be monitored for fungal infections, especially those exposed to postnatal glucocorticoids. Additionally, medical personnel should improve the prevention of fungal infections. Fluconazole prophylaxis has demonstrated significant efficacy in preventing invasive *Candida* infections in preterm infants. It is safe and resistance has not emerged when using 3–6 mg/kg twice a week^12–14,33^. This is the guideline for the prophylactic use of fluconazole in the clinical setting.

The present study indicates risk factors for positive PICC tip cultures in preterm infants. The findings may shed some light on preventing fungal colonization in premature infants in future clinical practice. However, our study also has some limitations. First, this was a retrospective study, and some bias might exist in the patient collection. Second, only a small number of patients from a single centre were enrolled in this study, which might affect the reliability of our study, and no significance was observed in some potential risk factors. Therefore, large sample multi-centre prospective cohort studies should be performed to validate our findings. In addition, it also should take into account that some risk factors may be affected by receiving 5 mg/kg of fluconazole prophylaxis twice a week while PICC line was in place in infants < 32 weeks or 1500 g.

In conclusion, in our study population, fungal colonization of the PICC tips was affected by the duration of antibiotic use, PN infusion, and postnatal glucocorticoid exposure. To prevent fungal colonization and further fungemia, clinical management strategies for the above risk factors should be improved.

## Materials and methods

### Study design and patient selection

This retrospective study was conducted between 1 January 2018 and 31 December 2020 at the Affiliated Hospital of Southwest Medical University. The enrolled infants who underwent ultrasound-guided PICC insertion during their hospitalization were born at < 32 weeks' gestation or birth weight < 1500 g. While the PICC line was in place, these enrolled infants were received 5 mg/kg of fluconazole prophylaxis twice a week. The blood culture and catheter tip culture were performed at the same time when the PICC catheters were removed. Exclusive criteria included accidental detachment, ectopic catheters, two or more catheterizations, and death before catheter removal.

The Institutional Review Board of the Affiliated Hospital of Southwest Medical University approved the study protocol (KY2021074) and waived informed consent because of the retrospective study design. We confirm that all methods were performed in accordance with the relevant guidelines and regulations.

### Procedural management of peripherally inserted central catheters

A 1.9-French silica gel PICC catheter was inserted into neonates by qualified nurses using ultrasound guidance. An X-ray was obtained to confirm the catheter tip's position in a central vein outside the cardiac silhouette. The catheterization room was a separate room, and all instruments including the air were disinfected after each catheterization. Dressings were changed 24 h after catheterization and once a week afterwards. However, if the dressings were wet, loose, or both, they were changed immediately. After removing the previous dressing, a skin disinfectant was used to disinfect the 10-cm^2^ area around the puncture site. The skin disinfectant comprised iodine of 0.2% ± 0.02%, chlorhexidine acetate of 0.45% ± 0.045%, and ethanol of 65% ± 5%. The catheter connector was replaced every week. Only syringes larger than 10 mL were used for drug infusion through the PICC. Aseptic technology was used for any puncture, fluid preparation and connection. The PICC was not used for blood draws or transfusions. Tubes were flushed with 2 mL of only saline every day before infusions were performed and sealed with 1 IU/mL of a heparin solution^34^. Health care workers continued to review infants’ needs for a PICC daily. The PICC was withdrawn when parenteral nutrient or hypertonic fluids were no longer required, or when CLABSI was highly suspected^34^.

### Definitions

Catheter colonization was defined as the distal part of the catheter having pathogens amounting to ≥ 15 colony-forming units (CFU)/tablet, with semiquantitative cultures or pathogens amounting to ≥ 1000 CFU on the quantitative culture. Five cm of the catheter tip was kept for pathogen culture after the PICC was removed immediately. At the time of PICC removal, 1–2 mL of blood was taken from the contralateral side of the PICC insertion limb for blood culture.

CLABSI was diagnosed using the following definition from the National Health Safety Net work: a laboratory-confirmed bloodstream infection in patients wherein an eligible bloodstream infection-causing organism was identified and an eligible central venous catheter was present on or one day before the infection date^35^.

Early-onset sepsis was defined as the presence of clinical symptoms and a positive culture from blood or cerebrospinal fluid samples drawn within 72 h of birth^36^.

### Data collection

Relevant clinical data from the hospital information system were separately collected using EpiData (version 3.02, EpiData Association) by two researchers. The collected data were as follows: demographics including sex, gestational age, birth weight, mode of delivery, assisted reproductive technology, twin pregnancy, and premature rupture of membranes; PICC-related characteristics including age at catheter insertion, indwelling time, tip position, and results of the PICC tip cultures; and treatment information including the duration of antibiotics, PN infusion, and glucocorticoid administration.

### Statistical analysis

Statistical analysis was performed using SPSS 19.0 (IBM Corp.). The categorical data were described as a percentage (%) and compared using the chi-square test. The continuous data with normal distribution were described as mean ± standard deviation and compared using the Student *t* test. The continuous data with abnormal distribution were described as a quartile and analysed using the rank-sum test. The variables with significant statistical differences were selected and included in the multivariate model. Because they had a strong collinear relationship, the PICC dwelling time and duration of PN were entered into the logistic regression model only one parameter at a time. Gestational age and birth weight were mentioned as important influencing factors in many works of literature^37–39^, so they were included in the model for statistical analysis. Then multivariate logistic regression was performed to calculate the adjusted odds ratios and 95% confidence intervals. The ROC curve was used to determine the optimal cut-off value for the duration of antibiotics and PN by calculating the Youden index.

## Data Availability

We state that the study data is available to readers.
